# Association of Mandibular Posterior Anatomic Limit with Skeletal Patterns and Root Morphology Using Three-Dimensional Cone Beam Computed Tomography Comprehensive Analysis

**DOI:** 10.3390/diagnostics12123019

**Published:** 2022-12-02

**Authors:** Keiichiro Iguchi, Yong-Il Kim, Mohamed Adel, Mohamed Nadim, Reina Hatanaka, So Koizumi, Tetsutaro Yamaguchi

**Affiliations:** 1 Department of Orthodontics, School of Dentistry, Kanagawa Dental University, 82 Inaoka-cho, Yokosuka 238-8580, Japan; 2 Department of Orthodontics, School of Dentistry, Pusan National University, 2, Busandaehak-ro 63beon-gil, Geumjeong-gu, Busan 46241, Republic of Korea; 3 Division of Orthodontics, College of Dentistry, University of Kentucky, Lexington, KY 40506, USA; 4 Department of Orthodontics, Suez Canal University, El Sheikh Zayed, EL SALAM DISTRICT, Ismailia 8366004, Egypt

**Keywords:** molar distalization, mandibular posterior anatomic limit, cone beam computed tomography, skeletal anteroposterior relations, vertical skeletal patterns

## Abstract

This study aimed to clarify the relationship between the mandibular posterior anatomic limit (MPAL) and skeletal anteroposterior and vertical skeletal patterns, with consideration of factors that may be related. In total, 230 people were included: 49 Japanese, 122 Egyptian, and 59 Korean people. The MPAL was measured at 0, 2, 4, and 6 mm from the root furcation along the sagittal and cuspal lines at the distance from the distal root of the mandibular right second molar to the mandibular cortex of the lingual bone. Eight different MPALs were evaluated using multiple regression analysis with explanatory variables for anteroposterior and vertical skeletal patterns and qualitative variables for age, sex, population, the presence of third molars, number of roots, presence of C-shaped roots, and Angle malocclusion classification. The MPAL was significantly larger as the mandibular plane angle decreased. The MPAL near the root apex was significantly larger as the A-nasion-point B angle increased, and the MPAL near the root apex measured at the cuspal line was significantly larger for C-type roots. The present study showed that a C-shaped root affected the MPAL in addition to the anteroposterior and vertical skeletal patterns.

## 1. Introduction

The mandibular posterior anatomic limit (MPAL) is limited by the distance from the distal root of the mandibular second molar to the mandibular cortex of the lingual bone [1]. Recently, with the development of temporary anchorage devices (TADs), treatment goals for malocclusion requiring distalization of the mandibular molar have become easier to achieve [1,2,3]. TAD is expected to provide predictable and effective tooth movement owing to its simplified mechanics, shorter treatment time, and the fact that it does not require patient cooperation [3]. Tooth movement occurs in the alveolar bone during dynamic treatment when appropriate orthodontic forces are applied to the teeth, resulting in bone remodeling and movement within the alveolar bone [4]. The thickness of the alveolar bone determines the limits of tooth movement during dynamic treatment, and exceeding these limits can cause damage to periapical tissues [3,5]. Evaluation of mandibular morphology and anatomical limitations during distalization is necessary to predict the risk of defects in the cortical bone, including loss and penetration during tooth movement, and to minimize the risk of root resorption, alveolar bone damage, and root exposure [1]. Therefore, the MPAL has long been studied using panoramic and lateral head radiographs [6]. However, this area has limitations in MPAL evaluation because of overlapping anatomical structures in two dimensions [1]. Conversely, cone beam computed tomography (CBCT) constructs images in three sizes and has superior spatial resolution compared to radiography; therefore, detailed information can be obtained using CBCT to evaluate the MPAL [7,8,9].

Recently, orthodontic clinical studies have examined the relationship between the MPAL and anteroposterior and vertical skeletal patterns. Kim et al. [10] classified patients with skeletal classification III malocclusion into low divergence (sella-nasion to gonion–gnathion angle ≤ 28.0°), normal divergence (28.0 < sella-nasion to gonion–gnathion angle ≤ 36.0°), and super divergence (36.0° ≤ sella-nasion to gonion–gnathion angle). The relationship between vertical skeletal patterns and the MPAL was examined by classifying them into the abovementioned categories. They reported that vertical skeletal patterns were highly correlated with the MPAL and that the MPAL showed a decreasing trend as the sella-nasion to gonion–gnathion angle increased. However, their study population did not include patients with skeletal class I or II malocclusion. Kim et al. [2] reported that normodivergent patients had a larger MPAL than hyperdivergent and hypodivergent patients and that the MPAL was associated with mandible size. Zhao et al. [11] reported that the MPAL is significantly smaller for hyperdivergent patients than for hypodivergent and normodivergent patients. However, in their study, only age and sex were considered as possible factors associated with skeletal anteroposterior and vertical skeletal patterns.

The relationship between skeletal anteroposterior relations and the MPAL was also examined by Choi et al. [12], who reported that in measurements at 0 mm and 2 mm from the bifurcation at the cuspal line, the MPAL was significantly greater in patients with skeletal class I malocclusion and that the MPAL was significantly greater in patients with skeletal class III malocclusion than in patients with skeletal class I malocclusion. However, Choi et al.’s study included a larger percentage of patients with skeletal class III malocclusion than skeletal class I malocclusion, did not include patients with skeletal class II malocclusion, compared patients with skeletal class I malocclusion and skeletal class III malocclusion, and did not examine comprehensive skeletal anteroposterior relations. Kim et al. [2] reported that the MPAL in patients with skeletal class I malocclusion was greater than that in patients with skeletal class II malocclusion and class III malocclusion, and that MPAL was reported to be influenced by the mandibular size and other factors. Kim et al. [2] considered the A-nasion-point B angle (ANB) (assessment of skeletal anteroposterior relations), face height ratio, and sex (assessment of vertical skeletal patterns) as factors but not other factors, including the third molar.

In addition, would it be appropriate to include distalization in the treatment plan tooth root morphology? C-shaped roots, sometimes seen in mandibular second molars, have been reported to have a narrowing morphology toward the root apex [13]. Additionally, because the distance limits the MPAL from the distal root of the mandibular second molar to the mandibular cortex of the lingual bone, the presence or absence of a C-shaped root may affect the size of the MPAL. However, no study has examined the relationship between root morphology and the MPAL.

This study aimed to identify the causes of the MPAL using explanatory variables and quantitative variables, including the mandibular plane angle (MP), for assessing the vertical pattern of the skeleton and ANB for assessing the anteroposterior relationship of the skeleton. In addition, we aimed to clarify the association between the MPAL and C-shaped roots using the following qualitative variables: age, sex, population, the presence/absence of a third molar, number of roots, and Angle classification of malocclusion in teeth with C-shaped roots. Lastly, this study aimed to clarify the association between the MPAL and the presence or absence of a C-shaped root (qualitative variable) in CBCT images.

## 2. Materials and Methods

### 2.1. Ethics Statements

This study was approved by the relevant ethics committees of each medical institution (Pusan National University, IRB PNUDH-2019-025; Suez Canal University, IRB 8; and Kanagawa Dental University Committee, 841). All subjects, including Japanese, Egyptian, and Korean patients, visited the orthodontist at each institution between 2014 and 2022 and had CBCT for diagnosis. The CBCT data used in this study were not obtained exclusively. Written informed consent was obtained from the subjects prior to participation in this study.

### 2.2. Study Design and Population

The inclusion criteria of this cross-sectional study were as follows: (1) age > 18 years; (2) no acute periodontitis or mandibular molar alveolar margins; and (3) no missing teeth except the third molars. Exclusion criteria were (1) a history of orthodontic treatment; (2) facial asymmetry (menton deviation > 3.0 mm) evaluated by frontal cephalography using the midsagittal reference plane formed by the crista galli, anterior nasal spine (ANS), and opisthion; (3) craniofacial syndrome; (4) prosthesis of any of the molars; (5) insufficient quality CBCT images due to artifacts; and (6) CBCT images with inadequate coverage. The characteristics of the included participants are shown in Table 1.

### 2.3. Imaging and Definitions

CBCT images of the Japanese population were obtained using a cone-beam X-ray computed tomography (CT) system (KaVo OP 3D Vision, Tokyo, Japan) with a voxel size of 0.3 mm. CBCT images of the Korean population were obtained using a cone-beam X-ray CT system (Zenith 3D; Vatech Co., Seoul, Republic of Korea) with a voxel size of 0.3 mm. CBCT images of the Egyptian population were acquired using a cone-beam X-ray CT system (Soredex SCANORA 3D; Nahkeatine 16, Tuusula, Finland) with a voxel size of 0.5 mm. Torres et al. [14] measured mandibular areas at 0.2, 0.3, and 0.4 mm and reported no differences between the voxels. Therefore, the voxel sizes used in this study were 0.3 mm and 0.5 mm; although 0.5 mm is a large voxel size, it did not affect the accuracy between voxels based on a previous report [15]. Maxillary and mandibular basilar arch skeletal anteroposterior relations including the ANB and MP (all angles), and MPAL distances were measured under standardized conditions using InVivo Dental 6 software (Anatomage Inc., San Jose, CA, USA). The MPAL is defined as the distance from the distal root of the mandibular right second molar to the mandibular cortex of the lingual bone [10], according to the method reported by Choi et al. [12]. The MPAL measurements were taken at 2 mm, 4 mm, and 6 mm apical to the root furcation along the root furcation and sagittal and cuspal lines [12].

**Figure 1:**
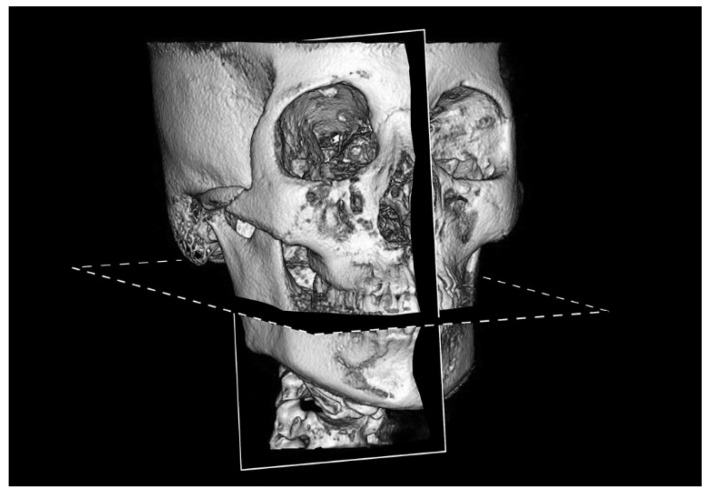
Reference planes used in this study. Midsagittal plane (solid line): the plane passing through the anterior nasal spine, crista galli, opisthion, and midpoint of the mandibular incisor tip. Mandibular occlusal plane (dotted line): the plane passing through the proximal buccal cusp of the mandibular first molar and the tip of the right mandibular central incisor.

Following Choi et al.’s method [12], we established a mandibular occlusal plane through the proximal buccal cusp of the mandibular first molar and the tip of the right mandibular central incisor, and a midsagittal plane through the ANS, crista galli, opisthion, and midpoint of the mandibular incisor tip (Figure 1).

**Figure 2:**
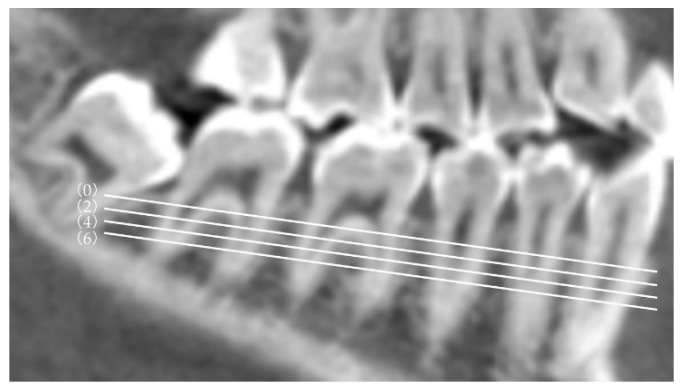
Plane used to measure the mandibular posterior anatomic limit to the mandibular cortex of the lingual bone. Reference plane (0) parallel to the mandibular occlusal plane passing through the root furcation of the mandibular right second molar. Reference planes (2), (4), and (6) are located 2, 4, and 6 mm, respectively, from reference plane (0) toward the root apex.

**Figure 3:**
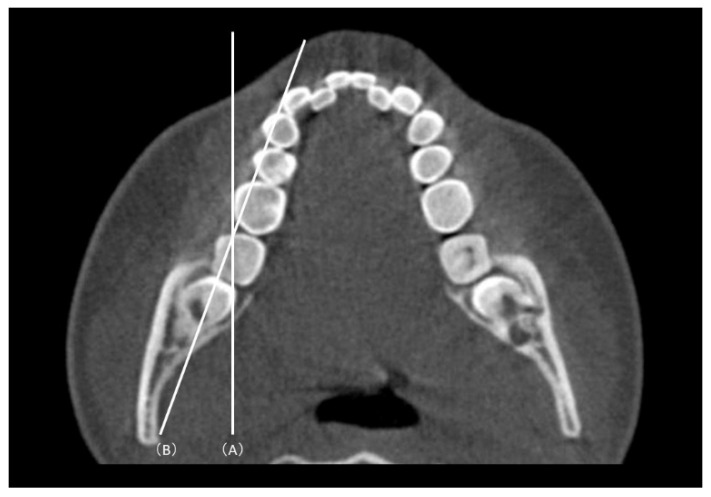
Reference lines used in this study. (A) Sagittal reference plane (the plane passing through the crista galli, menton anterior nasal spine, and opisthion). (B) The cuspal line connecting the buccal cusp of the first molar and mandibular second molar.

**Figure 4:**
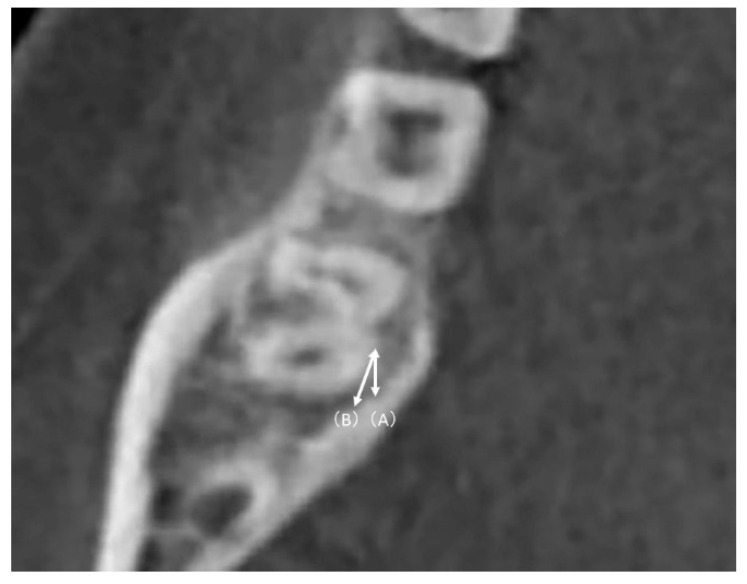
Definition of the mandibular posterior anatomic limit as measured in this study. Distances from the most lingual point of the distal root of the right mandibular second molar: (A) Distance from the medial side of the mandibular cortex of the lingual bone along the sagittal plane. (B) Distance from the medial margin of the mandibular cortex of the lingual bone along the cuspal line.

The plane passing through the root furcation of the mandibular second molar parallel to the mandibular occlusal plane was designated as plane 0; three planes parallel to the 0 plane and located 2, 4, and 6 mm toward the root apex were designated as planes 2, 3, and 4, respectively (Figure 2). Two additional reference lines were used to measure the MPAL: a sagittal line parallel to the midsagittal reference plane and a cuspal line passing through the buccal cusp of the left and right mandibular first and second molars (Figure 3). The shortest straight-line distance (MPAL) from the most lingual point of the distal root of the mandibular right second molar to the mandibular cortex of the lingual bone was measured (Figure 4).

### 2.4. Data and Statistical Analysis

We performed a power analysis using G*power (version 3.1.9.7; Franz Faul, Christian-Albrechts-Universitat, Kiel, Germany) and estimated power using a sample size of 230. When the conditions were set to an effect size of 0.15, total sample size of 230, and significance level of 0.05, a power (1-β) of 0.989 was obtained. Therefore, we considered that the sample size was sufficient.

CBCT images were used to evaluate skeletal anteroposterior relations using the ANB and vertical skeletal patterns by the MP. Multiple regression analysis (forced entry method) of eight MPALs was performed using the ANB and MP as explanatory variables. To evaluate measurement error, the MPAL of a randomly selected sample of 40 individuals was measured twice under the same conditions, and the error was tested using the Dahlberg formula [16,17]. The method errors ranged from 0.270 to 0.940 mm. The statistical analysis was performed using the statistical package SPSS Statistics (version 25.0; IBM Corporation, Armonk, NY, USA).

The paired *t*-test showed no statistically significant differences between repeated measurements (Table 2).

The raters were very reliable across repeated measures. Each of the eight measures of root furcation and 2, 4, and 6 mm from the root furcation along the sagittal and cuspal lines were used as a single objective variable. The presence or absence of third molars, number of roots (one or two), presence or absence of C-shaped roots, ANB, MP, and Angle classification of malocclusion were used as multiple explanatory variables in multivariate analysis (multiple regression analysis). Regression analysis was performed to determine explanatory variables that were significantly related to the objective variable. Age, ANB, and MP were continuous variables of the factors used as explanatory variables, whereas the other factors were used as categorical data in multiple regression analysis. The significance level was set at α = 0.05 (two-tailed), with a *p*-value < 0.05 indicating a significant difference. Minitab 19 (Minitab LLC, State College, PA, USA) was used to perform the statistical analysis. The usual multiple regression analysis models could not be applied in this analysis because the explanatory variables included categorical data. Therefore, Minitab, which is capable of flat processing, was used.

## 3. Results

The results of the multiple regression analysis are shown in Table 3 and Table 4. The smaller the MP, the larger the MPAL was for all measurement points up to 6 mm from the root furcation measured at the sagittal and cuspal lines, even after taking into account the age, sex, population, presence or absence of the third molar, number of roots, Angle’s classification of the malocclusion, and the presence or absence of a C-shaped root. The larger the ANB, the larger the MPAL was for all measurement points up to 4 mm from the root furcation measured at the sagittal and cuspal lines. Females showed significantly smaller MPAL values at 2 mm from the root furcation as measured at the sagittal and cuspal lines. Egyptian subjects had a significantly higher MPAL at the root furcation measured at the cuspal line. If the third molars were present, MPAL was significantly higher at 2 mm from the root furcation measured at the sagittal line and at 2 mm from the root furcation measured at the sagittal and cuspal lines. If the mandibular second molar had a C-shaped root, the MPAL was significantly greater at 4 mm and 6 mm from the root furcation measured at the cuspal line.

## 4. Discussion

Clarifying the relationship between the MPAL and anteroposterior and vertical skeletal patterns, which was the objective of this study, will allow predictive and effective orthodontic treatment [12]. When distalization is performed, the positional relationship between the root and cortical bone must first be confirmed [18]. Distalization is a form of orthodontic treatment performed to avoid tooth extraction in patients with skeletal abnormalities [19]. In particular, skeletal abnormalities have been reported to deviate from normal in the thickness of the mandibular cortex of the lingual bone and morphology of the mandibular buccal alveolar processes [20,21]; thus, it is important to have an accurate understanding of skeletal morphology. C-shaped roots, sometimes seen in mandibular second molars, have been reported to have a variety of forms [13] and may be related to the MPAL. Therefore, we investigated this possibility. In addition to anteroposterior and vertical skeletal patterns, this study revealed that the presence or absence of a C-shaped root affects the MPAL.

Our study showed that the smaller the MP, the larger the MPAL for all measured items up to 6 mm from the bifurcation, measured at the sagittal and cuspal lines. Kim et al. [2] examined the MPAL of Korean patients classified into anteroposterior skeletal patterns and vertical skeletal patterns. Their results differ from those of the present study. The reason for the difference may be that we evaluated vertical skeletal patterns in terms of the MP, while Kim et al. [2] evaluated vertical skeletal patterns in terms of the face height ratio. Additionally, their study examined only sex as a factor that may be related to anteroposterior and vertical skeletal patterns. Furthermore, Kim et al. [10] studied the MPAL in Korean patients with skeletal class III malocclusion who were classified into three categories of vertical skeletal patterns (hypodivergent, normodivergent, and hyperdivergent). Their study population did not include patients with skeletal class I or class II malocclusion. However, their results were similar to those of the present study. Zhao et al. [11] studied the MPAL in Chinese patients with skeletal class I malocclusion and classified them into three categories of vertical skeletal patterns (hypodivergent, normodivergent, and hyperdivergent). Vertical skeletal patterns were highly correlated with the MPAL, and hyperdivergent patients were reported to have a significantly smaller MPAL than hypodivergent and normodivergent patients. However, their study population did not include patients with skeletal class II or class III malocclusion. Nevertheless, their results were similar to those of the present study. Hypodivergent patients have greater occlusal forces [2,10,22] and increased attachment of the mandibular lingual cortical bone, whereas hyperdivergent patients have a narrower and thinner mandible [21]. Ingervall et al. [22] examined the cranial lengths of patients with both strong and weak occlusion on standardized head X-rays. Occlusal force is related to vertical skeletal patterns; hypodivergent patients have a stronger masticatory force and a larger mandibular body length and diameter than hyperdivergent patients [23]. Some genes associated with long faces may be involved in the determination of the MPAL [24]. Therefore, this study’s results are valid, as differences in vertical skeletal patterns have been shown to be associated with the MPAL via occlusal force and the mandibular body length [2,10]. The present study’s results showed that the larger the ANB, the significantly larger the MPAL at 4 mm from the bifurcation when measured at the sagittal and cuspal lines. Choi et al. [12] reported that the MPAL measured using CBCT near the crown (0 mm and 2 mm depth to the branch) was significantly larger in patients with skeletal class III malocclusion than in those with skeletal class I malocclusion. However, their study did not include patients with skeletal class II malocclusion, did not compare patients with skeletal class I malocclusion with those with skeletal class III malocclusion, and did not examine comprehensive skeletal anteroposterior relations. Kim et al. [2] reported that the MPAL was greater in patients with skeletal class I malocclusion than in those with skeletal class II malocclusion and skeletal class III malocclusion, however, these differences were not statistically significant. Kim et al. measured the area divided into three sections (the furcation area, center, and root apex), whereas we measured the area divided by four planes located at 2, 4, and 6 mm from the furcation area in the apex direction. Therefore, the measured areas were different, and the MPAL values may be dependent on the length of the root, which may explain the difference between the results of the previous study (Kim et al.) and those of the present one. Hwang et al. [25] classified 143 Korean patients into skeletal classes I, II, and III and used CBCT to study the relationship between the buccolingual tilt of the mandibular molar and ANB. The results showed a significant positive correlation between the ANB and the buccal tilt of the mandibular molar. The root apex of the tooth is often in a more normal position than the crown of the tooth, whereas the root of the crown of the tooth is located more buccally as the ANB increases. The MPAL increased accordingly. Coşkun et al. [19] also examined cortical and trabecular bone thickness from CBCT images of the upper and lower jaw of 201 Turkish patients classified as skeletal classes I, II, and III. The results showed no significant differences in cortical bone thickness between the three groups. However, the trabecular bone thickness was significantly greater in the maxillary and mandibular tooth regions in patients with skeletal class II malocclusion than in those with skeletal class I and III malocclusion. This is because patients with skeletal class II malocclusion have a buccally inclined crown, which may cause the root to be inclined, resulting in bone formation and larger alveolar bone space. Ribeiro et al. [26] also reported that patients with skeletal class III malocclusion had a decreased proximal buccal width diameter of the mandibular branch compared with patients with skeletal class II malocclusion. Thus, this study’s results may explain the validity of the significantly larger MPAL values up to 4 mm from the bifurcation, as measured at the sagittal and cuspal lines, as the ANB increases. Patients with skeletal class III malocclusion have a narrower alveolar bone space and reduced proximal buccal width of the mandibular branch than patients with skeletal class II malocclusion. Patients with skeletal class II malocclusion may have a bone morphology that is difficult to distalize. By contrast, anteroposterior skeletal patterns may be involved in MPAL. In Kim et al.’s study [10], the distribution of vertical skeletal patterns was 33.3% each, and they found that hypodivergent patients accounted for 46.5% of the population, hyperdivergent patients for 25.4%, and normodivergent patients for 28.1%. Here, the distribution was similar to that reported by Kim et al. [2]: hypodivergent patients, 41.3%; hyperdivergent patients, 22.6%; and normodivergent patients, 36.1%. Thus, the distribution of vertical skeletal patterns is different in each case, suggesting that the MPAL may be strongly related to vertical skeletal patterns rather than anteroposterior skeletal patterns. The MPAL may be more likely to involve vertical skeletal patterns than anteroposterior patterns.

This study also showed that patients with third molars have a significantly larger MPAL near the tooth crown. This finding replicates the results of a previous study [27]. By contrast, Kim et al. [1] reported that the presence or absence of third molars in the mandible did not affect the MPAL in patients with skeletal class III malocclusion. This difference in results may indicate that the presence or absence of third molars may not be accurately diagnosed as congenitally deficient or due to extracted teeth [6,10,27].

There was no association between the number of roots and the MPAL. The MPAL was significantly higher when the root of the mandibular second molar was a C-shaped root at 4 mm and 6 mm from the root furcation measured by the cuspal line (Table 4). This is the first study to show a relationship between the MPAL and C-shaped root.

Although the mandibular second molar has a smaller root divergence angle than the mandibular first molar, the mandibular second molar is usually closer to the mandibular cortex of the lingual bone. C-shaped roots have a narrowing of the root in the tooth root apex direction, although there are several types [13]. These results suggest that the C-shaped root affected the MPAL. Therefore, the present study revealed that in addition to anteroposterior and vertical skeletal patterns, the presence or absence of a C-shaped root affects the MPAL.

The Angle classification of malocclusion was not a significant factor for the MPAL. Similarly, no statistically significant difference was found in Chen et al.’s report [27]: the MPAL was not associated with the Angle classification of the malocclusion, the proximal centrifugal position of the maxillary first molar [2,10,12,26], and depended on the buccolingual position of the mandibular molar [24] and the morphology of the mandibular body [2,10], which was also revealed here.

One limitation of this study is that the voxel size used for CBCT images in the Egyptian population was 0.5 mm, which is inaccurate [28,29]. Torres et al. [14] reported no difference between voxel sizes of 0.2, 0.3, and 0.4 mm in the evaluation of linear measurements. Therefore, the voxel size of 0.5 mm used for the CBCT images did not affect this study’s results. In addition, embedding the centrum gingiva during the distalization of the mandibular dental arch may be another limitation [10,26]. An evaluation method that would allow the width and thickness of the central gingiva of the mandibular second molar to be included in the study is needed. An evaluation method is expected to be developed such that the width and thickness of the mandibular second molar can be considered.

## 5. Conclusions

Being female, being Caucasoid (such as being Egyptian), and having the third molar on the distalization side may affect the limits of distalization of the mandibular dental arch. In addition, the root morphology of the mandibular second molars, the prominent MP in hyperdivergent patients, and the small ANB in skeletal class III patients may affect the limits of distalization of the mandibular dental arch. When considering distalization in treatment planning, it is important to evaluate the relationship between the MPAL and anteroposterior and vertical skeletal patterns by using CBCT imaging. This study revealed that, in addition to anteroposterior and vertical skeletal patterns, the presence or absence of a C-shaped root affects the MPAL.

## Figures and Tables

**Table 1:** Characteristics of the participants.

	Japanese n = 49	Egyptians n = 122	Koreans n = 59
Sex			
Male	10	49	29
Female	39	73	30
Third molars			
Yes	27	100	26
No	22	22	33
Number of roots			
1	22	7	17
2	27	115	42
C-shaped root			
Yes	16	11	21
No	33	111	38
Malocclusion			
Angle class I	13	78	28
Angle class II	31	23	7
Angle class III	5	21	24

**Table 2:** Analysis of error of the method using the paired *t*-test and Dahlberg’s formula.

	First Measurement	Second Measurement	*p*-Value	Casual Error
A-0	4.515 ± 1.768	4.507 ± 1.857	0.920	0.369
A-2	4.100 ± 1.620	4.016 ± 1.673	0.257	0.327
A-4	3.770 ± 1.327	3.660 ± 1.323	0.066	0.270
A-6	3.345 ± 1.371	3.217 ± 1.438	0.064	0.311
B-0	8.240 ± 3.256	7.982 ± 3.138	0.223	0.940
B-2	6.935 ± 2.659	6.973 ± 3.061	0.791	0.635
B-4	6.060 ± 2.313	5.892 ± 2.332	0.068	0.414
B-6	5.100 ± 2.412	5.019 ± 2.382	0.464	0.490

**Table 3:** Results of multiple regression analysis (sagittal line).

Response Variable	Explanatory Variable	Β	95% Confidence Interval for Β		β	*p*-Value	
			Lower	Upper			
A-0	Age	0.057	−0.014	0.128	0.102	0.116	
	ANB	0.151	0.050	0.252	0.232	0.004	**
	MP	−0.094	−0.145	−0.043	−0.264	0.000	***
	Sex	−0.651	−1.324	0.022	−0.123	0.058	
	Egyptian	0.250	−0.235	0.736	0.097	0.311	
	Korean	−0.173	−0.512	0.166	−0.088	0.315	
	Third molar	0.797	0.091	1.502	0.146	0.027	*
	Number of roots, one or two	−0.071	−1.432	1.290	−0.011	0.919	
	C-shaped root	1.024	−0.255	2.304	0.163	0.116	
	Angle class II	−0.329	−0.734	0.076	−0.113	0.111	
	Angle class III	0.002	−0.291	0.296	0.001	0.987	
A-2	Age	0.034	−0.031	0.099	0.070	0.298	
	ANB	0.111	0.019	0.204	0.194	0.018	*
	MP	−0.073	−0.120	−0.026	−0.233	0.002	**
	Sex	−0.683	−1.299	−0.066	−0.147	0.030	*
	Egyptian	0.117	−0.327	0.562	0.052	0.604	
	Korean	−0.184	−0.494	0.126	−0.107	0.243	
	Third molar	0.487	−0.159	1.132	0.102	0.139	
	Number of roots, one or two	−0.216	−1.462	1.030	−0.038	0.733	
	C-shaped root	0.837	−0.334	2.008	0.152	0.160	
	Angle class II	−0.207	−0.578	0.163	−0.081	0.271	
	Angle class III	−0.019	−0.287	0.250	−0.010	0.892	
A-4	Age	0.034	−0.029	0.096	0.073	0.289	
	ANB	0.090	0.001	0.179	0.167	0.047	*
	MP	−0.060	−0.105	−0.015	−0.205	0.009	**
	Sex	−0.560	−1.152	0.033	−0.129	0.064	
	Egyptian	0.063	−0.364	0.490	0.030	0.771	
	Korean	−0.145	−0.443	0.153	−0.090	0.339	
	Third molar	0.438	−0.182	1.059	0.098	0.165	
	Number of roots, one or two	−0.146	−1.343	1.052	−0.028	0.811	
	C-shaped root	0.790	−0.335	1.916	0.153	0.168	
	Angle class II	−0.098	−0.454	0.258	−0.041	0.588	
	Angle class III	−0.002	−0.260	0.256	−0.001	0.986	
A-6	Age	0.010	−0.047	0.067	0.025	0.724	
	ANB	0.067	−0.014	0.148	0.139	0.105	
	MP	−0.050	−0.091	−0.009	−0.191	0.016	*
	Sex	−0.298	−0.839	0.243	−0.076	0.279	
	Egyptian	0.024	−0.366	0.414	0.012	0.905	
	Korean	−0.155	−0.427	0.117	−0.107	0.262	
	Third molar	0.314	−0.253	0.881	0.078	0.276	
	Number of roots, one or two	0.174	−0.919	1.268	0.037	0.754	
	C-shaped root	0.915	−0.113	1.942	0.197	0.081	
	Angle class II	0.108	−0.217	0.433	0.050	0.512	
	Angle class III	0.058	−0.177	0.294	0.038	0.627	

Sex (0, male; 1, female), Egyptian, Korean, third molar, number of roots, C-shaped root, Angle class II, and Angle class III were simultaneously entered as dummy variables. * *p* < 0.05; ** *p* < 0.01; *** *p* < 0.001. ANB, A-nasion-point B angle; MP, mandibular plane angle.

**Table 4:** Results of multiple regression analysis (cuspal line).

Response Variable	Explanatory Variable	Β	95% Confidence Interval for β		β	*p*-Value	
			Lower	Upper			
B-0	Age	0.092	−0.033	0.218	0.092	0.149	
	ANB	0.225	0.045	0.404	0.192	0.014	*
	MP	−0.147	−0.237	−0.056	−0.229	0.002	**
	Sex	−1.138	−2.334	0.058	−0.120	0.062	
	Egyptian	1.014	0.152	1.877	0.219	0.021	*
	Korean	−0.090	−0.692	0.512	−0.025	0.769	
	Third molar	1.702	0.449	2.955	0.174	0.008	**
	Number of roots, one or two	0.161	−2.257	2.579	0.014	0.895	
	C-shaped root	1.892	−0.380	4.165	0.168	0.102	
	Angle class II	−0.400	−1.119	0.319	−0.076	0.274	
	Angle class III	0.199	−0.322	0.720	0.053	0.452	
B-2	Age	0.060	−0.048	0.169	0.071	0.274	
	ANB	0.216	0.062	0.371	0.219	0.006	**
	MP	−0.131	−0.210	−0.053	−0.243	0.001	**
	Sex	1.048	0.015	2.081	0.131	0.047	*
	Egyptian	1.275	−0.215	2.765	0.163	0.093	
	Korean	−0.278	−1.837	1.281	−0.031	0.725	
	Third molar	1.234	0.151	2.316	0.149	0.026	*
	Number of roots, one or two	0.419	−1.669	2.508	0.043	0.693	
	C-shaped root	1.819	−0.143	3.782	0.191	0.069	
	Angle class II	−0.361	−1.602	0.881	−0.041	0.567	
	Angle class III	0.500	−0.850	1.851	0.053	0.466	
B-4	Age	0.049	−0.048	0.145	0.067	0.320	
	ANB	0.168	0.031	0.305	0.198	0.017	*
	MP	−0.109	−0.179	−0.040	−0.236	0.002	**
	Sex	0.786	−0.129	1.701	0.114	0.092	
	Egyptian	0.560	−0.759	1.879	0.084	0.404	
	Korean	−0.498	−1.878	0.883	−0.065	0.478	
	Third molar	0.895	−0.063	1.854	0.126	0.067	
	Number of roots, one or two	0.552	−1.298	2.401	0.066	0.557	
	C-shaped root	1.834	0.096	3.573	0.225	0.039	*
	Angle class II	−0.306	−1.405	0.794	−0.040	0.584	
	Angle class III	0.420	−0.776	1.616	0.052	0.490	
B-6	Age	0.035	−0.051	0.122	0.056	0.421	
	ANB	0.074	−0.050	0.197	0.099	0.240	
	MP	−0.085	−0.148	−0.023	−0.210	0.007	**
	Sex	0.503	−0.321	1.326	0.083	0.230	
	Egyptian	0.352	−0.836	1.540	0.060	0.560	
	Korean	−0.482	−1.725	0.761	−0.072	0.446	
	Third molar	0.818	−0.045	1.681	0.131	0.063	
	Number of roots, one or two	0.368	−1.297	2.033	0.050	0.664	
	C-shaped root	1.747	0.182	3.313	0.244	0.029	*
	Angle class II	0.270	−0.720	1.260	0.041	0.591	
	Angle class III	0.125	−0.952	1.202	0.017	0.820	

Sex (0, male; 1, female), Egyptian, Korean, third molar, number of roots, C-shaped root, Angle class II, and Angle class III were simultaneously entered as dummy variables. * *p* < 0.05; ** *p* < 0.01. ANB, A-nasion-point B angle; MP, mandibular plane angle.

## Data Availability

The data presented in this study are available from the corresponding author upon request.
